# Differences in Protein Expression and Gene Amplification of Cyclins between Colon and Rectal Adenocarcinomas

**DOI:** 10.1155/2009/285830

**Published:** 2009-12-15

**Authors:** Rolf Aamodt, Kristin Jonsdottir, Solveig Norheim Andersen, Johan Bondi, Geir Bukholm, Ida R. K. Bukholm

**Affiliations:** ^1^Faculty Division Akershus University Hospital, University of Oslo, 1474 Nordbyhagen, Norway; ^2^Department of Surgery, Akershus University Hospital, 1478 Loerenskog, Norway; ^3^EpiGen, Faculty Division Akershus University Hospital, University of Oslo, 1478 Loerenskog, Norway; ^4^Department of Pathology, Akershus University Hospital, 1478 Loerenskog, Norway; ^5^Department of Health Promotion, Akershus University Hospital, 1478 Loerenskog, Norway

## Abstract

Adenocarcinomas of rectum and colon may be different with regard to the cellular biological basis for cancer development. A material of 246 rectal cancers removed surgically at Akershus University Hospital in the years 1992–2000 was investigated and was compared to a material of 219 colon cancers operated on at Akershus University Hospital during the years 1988, 1990 and 1997–2000. There were highly significant differences between the rectal and the colon cancers in the protein expression of cyclin D1, cyclin D3, cyclin E, nuclear *β*-catenin, and c-Myc and in gene amplification of cyclin A2, cyclin B1, cyclin D1, and cyclin E. Gene amplification and protein expression in the rectal cancers correlated significantly for the cyclins B1, D3, and E. A statistically significant relation was observed between overexpression of cyclin A2 and local relapse of rectal carcinomas, as higher expression of cyclin A2 was associated with lower local recurrence rate.

## 1. Introduction

Despite the fact that adenocarcinomas of rectum and colon have the same appearance both macroscopically and microscopically, they may be different with regard to the cellular biological basis for cancer development. In a previous study, we found a higher protein expression of nuclear *β*-catenin in rectal cancers than in colon cancers [1], indicating biological differences between rectal and colon adenocarcinomas. Since *β*-catenin is involved in cell proliferation, it is important to evaluate whether other genes, involved in cell cycle and cell proliferation, may also be differentially expressed in these two carcinomas. 

Cyclins regulate cell cycle by binding to and activating cyclin dependent kinases (CDKs). CDKs in turn phosphorylate other proteins. Stimulation from cyclins is mandatory for cell cycle progression and cell division. Different cyclins act at different stages of the cell cycle. Cyclin D1, D2, and D3 bind to CDK4 and CDK6. This allows the cell to enter the G1 phase [2]. The expression of cyclin D does not vary through the cell cycle, in contrast to the expression of other cyclins [3]. Cyclin E binds to CDK2 and lets the cell go from G1 to S phase [4]. Cyclin A acts at two different steps during the cell cycle. Firstly, it binds to CDK2 and is necessary during S phase [5, 6]. Secondly, it binds to CDK1 and so permits the cell to enter M phase [7]. Cyclin B binds to CDK1 and lets the cell cycle progress through M phase [8, 9].

Abnormally located *β*-catenin plays its role in colorectal tumorigenesis [10] by being part of the signaling cascade of the Wnt pathway [11]. *β*-catenin acts in association with adenomatous polyposis coli (APC) in tumorigenesis [12, 13]. Mutations in either *β*-catenin or APC can distort the normal tumor suppressive effect of APC [13]. Somatic mutations of the APC gene cause malfunctioning APC in 80% of colorectal cancers [10]. This malfunctioning APC fails to reduce the level of cytoplasmic *β*-catenin [10, 14]. The resulting increased level of cytoplasmic *β*-catenin induces transcription of cyclin D1 and c-Myc through the TCF/LEF pathway [10].

c-Myc binds to specific binding sites on DNA, there regulating the transcription of other genes [15]. The protein expression of c-Myc is elevated in colon cancers [16, 17]. c-Myc is one of the most often deregulated oncoproteins in cancer [15, 18].

Overexpression of cyclins is associated with poor patient prognosis in colorectal cancer patients. One of the mechanisms behind overexpression is gene amplification of the cyclins' genes at the DNA level. However, the overexpression may also be caused at the posttranslational level. The prognostic value of cyclin overexpression may be different whether overexpression is caused by gene amplification or by impaired degradation. This may especially be important regarding cyclin A.

The clinical outcome of rectal cancer patients has historically been different from that of colon cancer patients. The most important difference has been the frequency of local relapse before irradiation was introduced as a routine preoperative treatment for a large proportion of the rectal adenocarcinomas. Local relapse is a result of “local expansion” of tumor cells, where cyclins may play an important role.

The aim of the present study was to evaluate whether there are differences in expression and gene amplification of cyclins in rectal compared to colon adenocarcinomas. We also wanted to relate our laboratory results on rectal cancers to patient prognosis.

## 2. Materials and Methods

### 2.1. Patient Materials

All available tumor samples from a consecutive series of 274 paraffin-embedded rectal adenocarcinomas removed surgically at Akershus University Hospital in the years 1992–2000 were scrutinized for inclusion into the survey. These surgical treatments were all primary operations. We decided to include solely tumors at a level of 15 centimeters (5.9 inches) or less from the anal verge (i.e., the outer border of the anus) (246 patients). This level is within the clinically commonly used and somewhat arbitrary range of 15 to 18 centimeters, used to define the border between rectum and colon. We wanted a restrictive border in order to avoid unintentional inclusion of sigmoid tumors.

This material of rectal carcinomas was compared to a material by Bondi et al. of 219 colon carcinomas operated on at Akershus University Hospital during the years 1988, 1990, and 1997–2000 [19].

Cancer specific death was registered only if metastasis was diagnosed, and the cancer disease was stated as the cause of death in the certificate of death.

Out of 246 patients 25 had a local recurrence of their rectal cancer. Mean time from primary operation was performed till local recurrence occurred, was 2.3 years. Minimum time was five months, maximum time 6.0 years. Mean observation time for patients with no local recurrence observed, was 6.2 years. Minimum observation time was 0 years (two patients), while maximum observation time was 14.5 years.

### 2.2. Immunohistochemistry

Serial sections (3-4 micrometers) from formalin fixed, paraffin wax embedded archive tumor tissue were applied to coated slides before immunohistochemical staining. After antigen retrieval by microwaving (20 minutes at 100°C), immunostaining with antibodies to cyclins A2, B1, D1, D3, and E, in addition to c-Myc and *β*-catenin, was performed, according to the operation protocols. Dako Autostainer (Dako Corporation, Carpinteria, CA) was used for cyclins B1, D1, E, c-Myc, and *β*-catenin, while staining for cyclins A and D3 was performed in Ventana ES automated slide stainer (Ventana Medical Systems, Tucson, AZ). The details of each antibody used are shown in Table 1. The antibodies were visualized for light microscopy with Envision Plus-System and diaminobenzidine (DAB), and with Detection Kit Ventana iView DAB, respectively. Counter staining was done with Hagen's haematoxylin for visualization of tissue structures. Positive control was a test block with normal colon mucosa and multiple colon adenocarcinomas with diverse differentiation.

**Figure 1:**
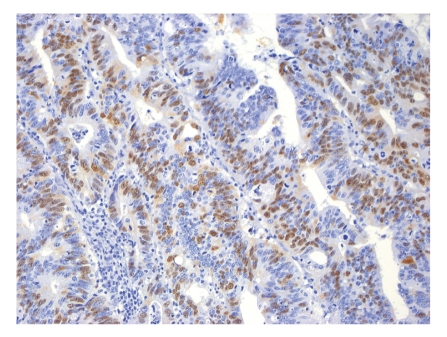
Representative example of immunopositivity grade 3 for cyclin A (original magnification ×200).

**Figure 2:**
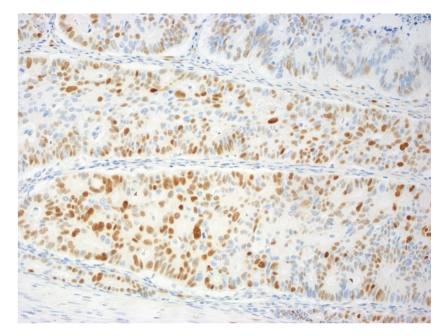
Representative example of immunopositivity grade 3 for cyclin D1 (original magnification ×200).

**Figure 3:**
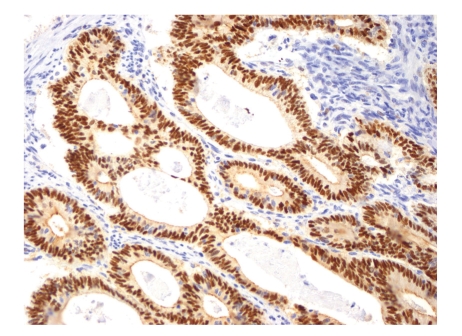
Representative example of immunopositivity grade 3 for cyclin E (original magnification ×200).

**Figure 4:**
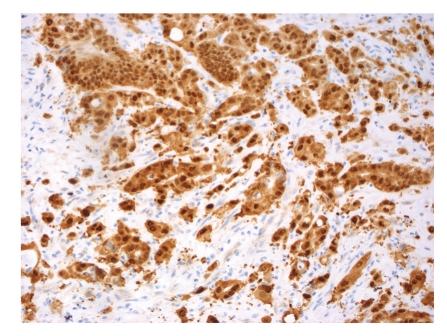
Representative example of immunopositivity grade 3 for *β*-catenin (original magnification ×200).

The percentage of positive nuclei was counted semiquantitatively by applying four grades of immunopositivity, 3, 2, 1, and 0. We intended to compare our findings on rectal cancers to findings previously recorded on colon cancers by Bondi et al. [19, 20]. We therefore chose the same cut-offs for the immunohistochemical markers as previously applied by them. When 60% or more of the tumor cell nuclei were stained, the tumor was graded as grade 3. Staining of 30% up to 60% of the nuclei was classified as grade 2. When nuclear staining was less than 30%, the score was grade 1. No nuclear immunostaining at all qualified for grade 0. Only clearly nuclear staining was recorded as positive, except in cyclin B1, where also cytoplasmic staining qualified for positivity [21]. Examples of immunostaining are shown in Figures 1, 2, 3, and 4. Almost all slides contained normal adjacent mucosa in addition to the cancer. The normal mucosa served as an internal control, and the level of cut-off was set at the staining level of normal mucosa. The slides were judged independently by three investigators (RAa, IRKB, and JB). At least 100, usually more than 1000 cells were examined in each slide.

### 2.3. Gene Amplification Analyses

Genomic DNA was extracted from 30 *μ*m section of formalin fixed, paraffin embedded (FFPE) tumor tissue, in a GenoMTM-48 Robotic Workstation (GenoVision, Oslo, Norway), as described by the manufacturer (GenoMTM-48, Qiagen protocol: Isolation of genomic DNA from paraffin-embedded sections using the MagAttract DNA Mini M48 Kit, December 2003, GenoVision). The DNA concentration was measured using NanoDrop ND-1000 Spectrophotometer (NanoDrop Technologies Wilmington, Delaware, USA). All samples consisted of >75% tumor tissue confirmed by light microscopy of a haematoxylin and eosin stained slide made from the adjacent tissue of the paraffin block.

To determine gene amplification or deletion we used real-time PCR on an ABI Prism 7900HT Sequences detection system (Applied Biosystems, Foster City, CA, USA) with software program SDS2.3. Primers and probes for cyclin B1, D1, D3, and E have previously been described by Bondi et al. [20]. Primers and probes for housekeeping gene human serum albumin (HSA) (assay Hs99999922_s1) were commercially designed by Applied Biosystems. Aagaard Sørby et al. designed primers and probes for cyclin A2 (personal communication). Primers and probe assays for cyclin A2 were purchased from Applied Biosystems. The order number was 2400949 in the Custom TaqMan Gene Expression Assay Service. The detailed information about primers and probes used for different cyclins is shown in Table 2.

We performed the PCR amplification of cyclins using a 96-well tray with a 20-*μ*L final reaction mixture containing 10 *μ*L TaqMan Universal PCR Master Mix, NoAmpErase UNG (2x), 1 *μ*L 20x Assay Mix, 4 *μ*L dH_2_O, and 5 *μ*L DNA (5 ng/*μ*L). For the rest of the cyclins, the PCR mix consisted of 2 *μ*L DNA (2–7.5 ng/*μ*L), 2x TaqMan Universal PCR Master Mix from Applied Biosystems, 600 nM of forward and reverse primer and 80 nM probes for the genes cyclin B1, D1, D3, and E according to Applied Biosystems manufacturer's instructions. The TaqMan gene expression assays for cyclin A2 and HSA contained 900 nM of forward and reverse primer and 250 nM FAM dye-labeled TaqMan probe. We added water to the total reaction volume of 20 *μ*L. All samples were run as triplicates. Default thermal cycling conditions were used in the PCR (Applied Biosystems).

To determinate the relative gene copy number of DNA of the cyclin genes we normalized the results to the level of HSA for each sample. This determination was done by means of the 2^−ΔΔCt^-method [22]. Threshold cycle number, Ct, for the real-time quantification was defined to be in the exponential phase of the PCR amplification. The calibrator used in the real-time PCR experiments was the mean of Ct-values of seven rectal cancer patients' normal rectal mucosal tissue.

### 2.4. Statistics

Statistical analyses were performed by SPSS version 14.0 running on Windows XP. Fisher's exact test, Kaplan-Meier log rank test, correlation analysis, binary logistic regression analysis, and Cox regression analysis were performed. We made test plots for proportional hazards for the Cox analyses and found them satisfactory. Pearson correlation was used when comparing protein expression to gene amplification. We chose an alpha level of statistical significance of *P* < .05.

## 3. Results

The clinico-histopathological characteristics of the patients are shown in Table 3.

There were highly significant differences between the rectal and the colon cancers in the protein expression of cyclin D1, cyclin D3, cyclin E, nuclear *β*-catenin, and c-Myc (Table 4), even when adjusted for Dukes' stage, differentiation grade, gender, and age of patient at the time of surgery.

There were also significant differences between rectal and colon cancers in gene amplification of all cyclins except cyclin D3 (Table 5), even when adjusted for gender, tumor differentiation grade, Dukes' tumor stage and age at surgery.

When gene amplification level of cyclins was evaluated in rectal adenocarcinomas, we observed the highest amplification level in the cyclin E gene, showing amplification in the largest proportion, 18%, of the samples. Cyclin A2 and cyclin D1 were amplified in 9% and 8%, respectively. The amplification proportion for cyclin D3 and cyclin B1 was only 2% and 1%, respectively (Table 6). The cut-off level was set to two.

When correlation between protein expression and gene amplification was analyzed, we observed a significant correlation between protein expression and gene amplification of cyclin B1, cyclin D3, and cyclin E, although the correlation coefficient was rather low (Table 7). No significant correlation existed for cyclin A2 or cyclin D1 (Table 7).

We examined associations between protein expression of different cyclins, as well as c-Myc and *β*-catenin, and patient prognosis in rectal cancer patients. In univariate analyses with Fisher's exact test (Table 8) there was a significant relation between occurrence of local recurrence and cyclin A protein expression (*P* = .0056 after Bonferroni correction). When we examined time from surgery till clinical manifestations in univariate analyses with Kaplan-Meier Log Rank Test (Table 9), there was a significant relation between local recurrence and cyclin A protein expression (*P* = .0062 after Bonferroni correction). We performed multivariate Cox regression analyses that included all seven examined proteins in addition to patient gender, tumor differentiation grade, Dukes' tumor stage, patient age at surgery, and time from surgery till clinical event. There was a significant association between cyclin A2 expression and reduced local relapse (*P* = .001, HR = 0.410, 95% CI for HR [0.247;0.682]). Expression of c-Myc was associated with reduced cancer specific survival in rectal cancer (*P* = .003, HR = 3.714, 95% CI for HR [1.568;8.797], while overexpression of c-Myc in colon adenocarcinomas not treated with adjuvant chemotherapy was associated with higher cancer specific survival (*P* = .028, HR = 0.353, 95% CI for HR [0.140;0.893]).

There was no significant relationship between gene amplification level in the rectal cancers and local recurrence of the cancer, distant metastases, cancer specific death, or lymph node metastases.

## 4. Discussion

In the present study, we have, for the first time, demonstrated that both protein expression and gene amplification of several proteins important for cell cycle progression, are different in rectal adenocarcinomas compared to colon adenocarcinomas, even when adjusted for Dukes' stage, tumor differentiation grade, and age of patient at the time of surgery. These results indicate different gene expression patterns and biological mechanisms between colon and rectal adenocarcinomas. The differences between these two entities observed in the present study can be correlated to clinical differences between the two diseases in clinical practice.

A statistically significant correlation was also observed between overexpression of cyclin A2 and local relapse of rectal carcinomas. Higher expression of cyclin A2 was associated with lower local recurrence rate. It is interesting to observe that the effect of cyclin A2 overexpression in rectal adenocarcinomas is different from its effect in most other cancers. Higher cyclin A2 expression may lead to poor patient prognosis for several other tumors, among them breast cancer [23–49]. So far, only in anal cancer [50] and probably in colon cancer cyclin A overexpression has indicated better clinical outcome.

Reports on the impact of cyclin A protein expression on clinical outcome of colon cancers diverge. Most of these studies find an unfavorable clinical effect in patients with high cyclin A protein expression in the tumor tissue. In two materials of mainly colon cancers and to a lesser extent rectal cancers regarded as a whole, cyclin A protein overexpression was associated with impaired overall survival [51, 52]. However, it is hard to decipher exactly how large proportion of one of these materials [52] was colon cancers. Another problem may be that overall survival may not reflect the cancer specific survival. In another material of colorectal patients the conclusion was the same [53]. These three reports were on colorectal patients, that is, a mixture of colon and rectal cancers. If the clinical effects of high cyclin A differ between colon and rectal cancers, the interpretation of these three studies might be somewhat difficult. In a material of solely colon cancers high cyclin A indicated better survival [20], especially in Dukes' stage D.

The expression of c-Myc was also analyzed in this study and compared to the expression of the same protein in colon adenocarcinomas. c-Myc stimulates cell growth [54]. Control of the cell cycle is usually lost if the expression of c-Myc is deregulated [55]. Higher c-Myc was associated with reduced cancer specific survival in rectal cancer patients, while in colon cancer patients without adjuvant chemotherapy, high c-Myc expression was associated with better prognosis.

To evaluate whether overexpression of cyclins detected in the present study was a result of amplification at the DNA level, gene amplification analyses were performed for all cyclins included in the study. Gene amplification analyses on the rectal cancers revealed a significant but fairly low correlation between gene amplification and protein expression only for the cyclins B1, D3, and E. No correlation between gene amplification and protein expression was observed for cyclin A2. Earlier studies have showed that protein overexpression of cyclins can occur without gene amplification [20, 56–58]. Our results indicate that gene amplification contributes to the variation seen in protein expression of cyclins in rectal cancer. But it does not offer a major explanation to this. Therefore, protein expression of cyclins in rectal cancer has to be regulated mainly by other mechanisms.

It is of importance, especially regarding cyclin A2, to explore the mechanism behind overexpression. It has been shown that overexpression of cyclin A in the S-phase of the cell cycle is associated with poor prognosis, while overexpression of cyclin A in the M-phase of the cell cycle is associated with better prognosis. It is reason to believe that overexpression caused by reduced degradation of a protein may result in accumulation of this protein also in the M-phase, while overexpression caused by high production, but intact degradation process may act mostly at the S-phase of the cell cycle. It is possible to speculate that overexpression of cyclin A2 caused by gene amplification may act mainly in the S-phase of the cell cycle. Thus, in rectal cancer, we believe overexpression of cyclin A2 protein is caused mainly by impaired degradation of the protein. This causes a high cyclin A2 concentration in M-phase in addition to in S-phase, leading to better clinical outcome.

Most colon cancers, especially those distal to the splenic flexure, evolve by the cromosomal instability (CIN) pathway. Fifteen percent of colon cancers evolve by the microsatellite instability (MSI) pathway. The MSI cancers are typically located proximal to the splenic flexure. In sporadic cancers, MSI is mainly caused by epigenetic silencing. There are indications of rectal cancers seldom having MSI [59] and often having CIN [60]. Other investigations only partially support these results [61, 62]. However, although their approaches differ from ours, except on *β*-catenin [62], individual results in these investigations indicate differences between rectal and colon cancers. This is in accordance with our findings.

## 5. Conclusions

In conclusion, only a few surveys exist on the biological differences between colon and rectal adenocarcinomas, despite the fact that colon and rectal cancer patients may have different clinical prognosis. The present study is one of the first studies where proteins important for cell cycle regulation are examined separately in colon and rectal adenocarcinomas. The results show biological differences between rectal and colon adenocarcinomas. The study demonstrates the necessity for examining these two disease entities separately. These biological differences may have significant impact when planning therapy for colorectal cancer patients.

## Figures and Tables

**Table 1:** Primary antibodies used for immunohistochemistry.

Antibody	Retrieval method	Dilution	Incubat time	Host species	Clone	Vendor
Cyclin A2	Tris/EDTA, pH 9	1 : 150	32 min	Mouse, monoclonal	6E6	Novocastra*
Cyclin B1	Tris/EDTA, pH 9	1 : 40	30 min	Mouse, monoclonal	7A9	Novocastra*
Cyclin D1	Dako TRS, pH 6	1 : 50	30 min	Rabbit, monoclonal	SP4	Lab Vision^†^
Cyclin D3	Tris/EDTA, pH9	1 : 20	30 min	Mouse, monoclonal	DCS-22	Novocastra*
Cyclin E	Tris/EDTA, pH9	1 : 40	30 min	Mouse, monoclonal	13A3	Novocastra*
c-Myc	Tris/EDTA, pH 9	1 : 50	30 min	Mouse, monoclonal	9E11	Novocastra*
*β*-catenin	Tris/EDTA, pH 9	1 : 300	30 min	Mouse, monoclonal	17C2	Novocastra*

*Novocastra, Newcastle, UK

^†^Lab Vision, Fremont, CA.

**Table 2:** Primer and probe sequences for cyclins used in real time quantitative polymerase chain reaction.

Gene	Primer sequence (5′–3′)	Hybridisation probe sequence (5′–3′)
CCNA2		
Forward	GCCACAGTAGGAGTTCTCCCATAT	FAM-CCCCGCCAACACTG-NFQ
Reverse	CAGACCGGCAGCATACACA	
CCNB1		
Forward	CCCTGCTGCAACCTCCAA	FAM-CCCGGACTGAGGCCAAGAACAGC-TAMRA
Reverse	TGTTCACTGACTTTGTTACCAATGTC	
CCND1		
Forward	CCGTCCATGCGGAAGATC	FAM-CCTCCAGCATCCAGGTGGCGA-TAMRA
Reverse	AACAAGTTGCAGGGAAGTCTTAAGA	
CCND3		
Forward	CTGTCTCTCCCCGCCAGTT	FAM-CACCCCCGACACGTATTGTCTCCC-TAMRA
Reverse	CTGATATCTCAAGCTTTCCTTTTCCT	
CCNE		
Forward	CCCCGCTGCCTGTACTGA	FAM-TCAGTGCCGACTCTGCCACATGG-TAMRA
Reverse	AGCATGGAGTAAGAGACCTGGAA	

**Table 3:** Rectal and colon cancers' characteristics, numbers (percentages in parentheses, (%)).

Rectal cancers				
Gender	Males: 150 (61)	Females: 96 (39)		
Age at operation	Lowest: 16	Mean: 66	Highest: 90	
Dukes' stage	A: 45 (18)	B: 100 (41)	C: 69 (28)	D: 30 (12)
T stage	T1: 8 (3)	T2: 46 (19)	T3: 187 (76)	T4: 5 (2)
N stage	N0: 154 (63)	N1: 63 (26)	N2: 29 (12)	
M stage	M0: 214 (87)	M1: 30 (12)		
Tumor differentiation	Poor: 7 (3)	Moderate: 235 (96)	High: 2 (1)	

Colon cancers				

Gender	Males: 105 (48)	Females: 114 (52)		
Age at operation	Lowest: 40	Mean: 70	Highest: 93	
Dukes' stage	A: 10 (5)	B: 105 (48)	C: 57 (26)	D: 47 (22)
T stage	T1: 4 (2)	T2: 27 (12)	T3: 173 (79)	T4: 14 (6)
N stage	N0: 137 (63)	N1: 65 (30)	N2: 15 (7)	
M stage	M0: 169 (77)	M1: 47 (22)		
Tumor differentiation	Poor: 23 (11)	Moderate: 184 (84)	High: 11 (5)	

**Table 4:** Protein expression. Variables that showed a significant difference between rectal cancers and colon cancers (results of binary logistic regression analysis on 400 patients).

Variable	Largest in rectum or colon	*P*-value	OR	95% CI for OR
Cyclin D1	Rectum	*P* < .001	9.933	[4.355; 22.654]
Cyclin D3	Colon	*P* < .001	0.239	[0.109; 0.524]
Cyclin E	Rectum	*P* < .001	3.282	[1.834; 5.872]
Nuclear *β*-catenin	Rectum	*P* < .001	38.514	[14.414; 102.906]
c-Myc	Colon	*P* = .001	0.253	[0.115; 0.558]

**Table 5:** Gene amplification. Variables that showed a significant difference between rectal cancers and colon cancers (results of binary logistic regression analysis on 403 patients).

Variable	Highest in rectum or colon	*P*-value	OR	95% CI for OR
Cyclin A2 amplification	Rectum	*P* < .001	23.286	[7.316; 74.119]
Cyclin B1 amplification	Rectum	*P* = .008	8.056	[1.718; 37.781]
Cyclin D1 amplification	Colon	*P* < .001	0.005	[0.001; 0.017]
Cyclin E amplification	Rectum	*P* < .001	5.248	[2.417; 11.395]
Age at surgery	Colon	*P* = .017	0.955	[0.919; 0.992]

**Table 6:** Amplification of cyclin genes of the rectal cancers.

Gene	*N* (total)	Amplification level*			
		<0.5	0.5–1.9	2.0–4.9	≥5
Cyclin A2	237	9	206	21	1
Cyclin B1	236	4	229	3	0
Cyclin D1	236	5	212	19	0
Cyclin D3	234	22	207	5	0
Cyclin E	235	2	191	40	2

*N-fold difference from the normal controls. Amplification was measured by real-time quantitative polymerase chain reaction, and levels were determined by the comparative C_t_ method (2^−ΔΔCt^ method).

**Table 7:** Correlation between protein expression and gene amplification of cyclins in rectal cancers.

Cyclin	Pearson correlation coefficient	*R* squared	*P*-value	Number of rectal cancers examined
Cyclin A2	0.101	0,010201	*P* = .124	232
Cyclin B1	0.156	0,024336	*P* = .017	233
Cyclin D1	−0.019	0,000361	*P* = .772	233
Cyclin D3	0.295	0,087025	*P* < .001	226
Cyclin E	0.188	0,035344	*P* = .004	232

**Table 8:** Cross table with Fisher's exact test. *P*-values without Bonferroni correction.

Protein	Local recurrence	Distant metastases	Lymph node metastases	Cancer specific death
Cyclin A	0.0008	0.299	0.286	0.141
Cyclin B	0.982	0.377	0.270	0.870
Cyclin D1	0.035	0.240	0.214	0.287
Cyclin D3	0.688	1.000	0.347	0.875
Cyclin E	0.481	0.522	0.075	0.088
Nuclear *β*-catenin	0.718	0.187	0.248	0.330
c-Myc	0.715	0.285	0.080	0.057

**Table 9:** Kaplan-Meier Log Rank Test. *P*-values without Bonferroni correction.

Protein	Local recurrence	Distant metastases	Cancer specific death
Cyclin A	0.000889	0.325	0.311
Cyclin B	0.854	0.425	0.692
Cyclin D1	0.055	0.295	0.322
Cyclin D3	0.575	0.964	0.734
Cyclin E	0.409	0.515	0.123
Nuclear *β*-catenin	0.528	0.172	0.274
c-Myc	0.281	0.269	0.082
